# Cleft palate, congenital heart disease, and developmental delay involving *MEIS2* heterozygous mutations found in the patient with attention deficit hyperactivity disorder: a case report

**DOI:** 10.3389/fped.2024.1500152

**Published:** 2024-12-24

**Authors:** Fang Shen, Junyan Li, Dandan Li, Hui Zhou

**Affiliations:** Key Laboratory of Birth Defects and Related Diseases of Women and Children of Ministry of Education (MOE), Department of Pediatrics, West China Second University Hospital, Sichuan University, Chengdu, Sichuan, China

**Keywords:** ADHD, *MEIS2*, cleft palate, congenital heart defect, developmental delay, case report

## Abstract

This case is the first reported patient with a *MEIS2* gene mutation who primarily exhibits pronounced inattention as the main manifestation and is diagnosed with ADHD, requiring methylphenidate treatment. It is characterized by unique clinical features that set it apart from previously reported cases with mutations in the *MEIS2* gene. Here, we report a female child with a diagnosis of ADHD and comorbidities. She received treatment with methylphenidate, starting at a dose of 18 milligrams per day, which was gradually increased to 45 milligrams per day based on her attention performance, while also undergoing physical and language rehabilitation training. In addition, the parents involved the child in reading and retelling stories at home every day. After 2 years of treatment, the scale results indicated that the child still had a moderate degree of attention deficit. Therefore, she underwent whole exome sequencing (WES) showing that her *MEIS2* gene carries a *de novo* frameshift mutation (c.934_937del, p. Leu312Argfs*11). After comparing the patient's features with those of other patients who also had the *MEIS2* mutation, we discovered that the patient's cleft palate, heart abnormalities, and minor facial dysmorphism were all extremely comparable. A broad forehead, elongated and arched eyebrows, and a tent-shaped upper lip were examples of mild facial dysmorphic traits. Subtypes with phenotypes such as cleft palate, cardiac anomalies, or facial malformations were presented in all previously reported cases of *MEIS2* mutations. Furthermore, less common characteristics include ADHD, learning difficulties, hearing loss, recurring respiratory infections, asthma, rhinitis, enuresis, and dental cavities. This case further supports the critical role of genetic testing in patients with ADHD who exhibit a suboptimal response to methylphenidate and present with multiple comorbidities. Furthermore, this case report expands the clinical symptom spectrum associated with *MEIS2* gene mutations, providing a broader understanding of the condition.

## 1 Introduction

ADHD is one of the most prevalent neurodevelopmental disorders, affecting approximately 5%–10% of children and adolescents worldwide (1). The disorder is characterized by persistent inattention, hyperactivity, and impulsivity, leading to significant challenges in all aspects of life, including academic performance, social interactions, and overall daily functioning (2). In addition, approximately 43% of childhood ADHD persists into adulthood (3). With regard to the cause of the disease, research suggests that it is due to the interaction of genetic and environmental factors, but that genetics play a considerable role. Based on the results of family and twin studies, the estimated heritability of ADHD approximates 80% (4, 5). The fact that ADHD frequently co-occurs with other diseases such as learning disabilities, anxiety disorders, and oppositional defiant disorder complicates both the diagnosis and treatment of ADHD. However, the advancement of genetic testing technologies has made it possible to determine the genesis of neurodevelopmental disorders exhibiting intricate clinical manifestations.

The *MEIS2* gene encodes a transcription factor belonging to the three-amino-acid-loop extension (TALE) protein superclass (6) and is an important transcription factor controlling embryonic development and cell differentiation. *MEIS2* is involved in the development of the heart and craniofacial region based on phenotypes linked to the overexpression or deletion of the gene in animal models (7–9), limb growth and patterning (10, 11), and axial skeletal patterning (12). The function of *MEIS2* in mouse palatal development has been thoroughly studied. Molecular and genomic analyses revealed that *MEIS2* directly regulates important osteogenic genes, and specific inactivation of the *MEIS2* gene in cranial neural crest cells resulted in complete cleft palate or submucous cleft and complete loss of palatal bone (9). In addition, Desiderio et al. (13) looked into the possibility that when crossing mouse strains to successfully deactivate *MEIS2* in the neural crest, the resulting cleft palate in the newborn pups would be consistent with earlier findings that *MEIS2* is essential for the development of the mouse's cranial and cardiac neural crest cells (7).

Additionally, *MEIS2* has a role in nearly every facet of the development of the central nervous system, such as neural tube patterning, proliferation of neural progenitor cells, acquisition of cell destiny, maturation of neurons, neurite outgrowth, and synaptogenesis (14–17), and is required for the survival and function of different neuronal populations (18, 19). Furthermore, *MEIS2* regulates the development of striatal neurons (20) and the maintenance of retinal progenitor cell pools (16). In the meantime, *MEIS2* is required for inner ear formation and proper morphogenesis of the cochlea (6).

At least 17 distinct mutations in the *MEIS2* gene have been linked to neurodevelopmental abnormalities in humans (21–23), highlighting the crucial role this gene plays in neuronal differentiation. Furthermore, *MEIS2* has been reported to be a susceptibility gene for obsessive-compulsive behaviors in humans. Somatic mutations that produce *de novo MEIS2* binding motifs are discovered in putative enhancer regions in the brains of people with autism spectrum disorders (24, 25). The characteristic features of a heterozygous missense mutation in the *MEIS2* gene locus or a 15q14 microdeletion are a triad of cleft palate, congenital heart defects, and intellectual disability, which is referred to as *MEIS2* syndrome (26).

This is the first report of an ADHD patient with a mutation in the *MEIS2* gene (c.934_937del, p. Leu312Argfs*11). Her primary clinical manifestations are attention deficit and developmental delay, and she also presents with issues involving multiple organ systems, including the respiratory, urinary, cardiac, oral, ear, and nasal systems. Additionally, her attention deficit did not improve significantly after treatment with methylphenidate. This case further extends the clinical phenotype of mutations in the *MEIS2* gene and confirms the importance of genetic testing in finding the etiology of ADHD in patients who do not respond well to methylphenidate treatment and have comorbidities.

## 2 Materials and methods

The study was approved by the Ethics Committee of West China Second Hospital of Sichuan University. In addition, written informed consent was obtained from the patient's parents before whole-exome sequencing was performed.

Peripheral blood samples (2–4 ml) from the pre-certified patient and her parents were collected into ethylenediaminetetraacetic acid (EDTA) anticoagulated blood sample tubes. Genomic DNA from this patient and parents was extracted from the blood according to the manufacturer's instructions (QIAamp DNA Blood Minikit). And WES was performed using the Illumina NovaSeq6000 platform. Sequencing reads were then aligned to the human reference genome GRCh38/HG38 and variants annotated using Burrows-Wheeler Aligner (BWA) software. Reads were aligned and locally recalibrated using GATK. A series of principles were used to screen for pathogenic mutations based on variant annotation, as follows: (1) Filtering for variants that are not seen to be carried by normal humans or have a carrier rate of less than 5% in databases such as gnomAD, 1,000 Genomes Project, and others. (2) Disease-causing mutation sites were evaluated using databases such as the Online Mendelian Inheritance in Man (OMIM) database, the National Institutes of Health (NIH), the Human Genetic Mutation Database (HGMD), and ClinVar. (3) Protein function prediction using software such as SIFT, PolyPhen2, and CADD. According to the American College of Medical Genetics and Genomics (ACMG) classification guidelines, the obtained single nucleotide variants (SNVs) were categorized into five categories, including pathogenic, possibly pathogenic, of uncertain significance, possibly benign, and benign. Finally, validation was performed using first-generation Sanger sequencing technology and samples from family members.

## 3 Case description

We report a 10-year-old girl who presented to the Department of Pediatric Rehabilitation, West China Second Hospital, Sichuan University, with what her parents described as inattention and developmental delay. The infant was born at 37 weeks of gestation, undersized for gestational age, weighing 2,100 g (below the 10th percentile), and measuring 47 cm (10th–25th percentile) in length. At birth, her parents noticed that she had a cleft palate, which was surgically corrected when she was one year old. A ventricular septal defect (VtSD), a patent foramen ovale (PFO), and mild tricuspid regurgitation were all detected by cardiac ultrasonography. Following that, the VtSD resolved on its own, and the foramen ovale has not yet closed. In addition, she developed full-mouth caries, asthma, rhinitis, enuresis, recurrent respiratory infections, and otitis media-related hearing loss. At the time of our initial evaluation, the child was 5 years and 2 months old, with a low bone age (equal to 4.9 years), a height of 105 cm (−2 SD), and a weight of 14.7 kg (between −2 and −3 SD). The patient also showed delayed motor development, beginning to walk at 18 months. She can no longer perform continuous movements, descends stairs slowly, and her coordination is weak. She also lags behind youngsters her age in fine motor skills, such as button fastening.

She also exhibits delayed language development. At 2 years and 10 months of age, she was only able to speak a few simple words, and her pronunciation was not accurate. Now at ten years old, her vocabulary in language communication is less than that of her peers, and she frequently experiences dysfluency in speech, which affects normal communication. During literacy reading, she skips words, and her logical thinking is poor. At school, multiple subject teachers have reported that she exhibits significant inattention during class, being able to concentrate for only about 20 min per lesson. Concurrently, she has learning difficulties and exhibits procrastination when doing homework, requiring parental supervision to complete tasks. Additionally, the child has poor social skills. Physical examination reveals mild facial dysmorphia, including a large forehead, extended and arched eyebrows, and a tent-shaped upper lip. These features are individually difficult to recognize.

The Wechsler Intelligence Scale for Children—Fourth Edition (WISC-IV) is used to assess children's level of intelligence, and the patient's total IQ score on the test was 92, which is at the normal level of intelligence for her age group. Based on the Diagnostic and Statistical Manual of Mental Disorders, Fifth Edition (DSM-5), the child was diagnosed with ADHD. The Swanson, Nolan, and Pelham, Version IV-26 items (SNAP-IV-26) consist of three parts: inattention, hyperactivity/impulsivity, and oppositional defiant. The scale uses a four-point scoring system ranging from 0 to 3, where 0 indicates the complete absence of such symptoms, and 3 represents the very frequent occurrence of these symptoms. It is used to assess the severity of ADHD symptoms, evaluate the effectiveness of treatment, and the degree of symptom improvement. The child showed moderately abnormal attention deficits using the SNAP-IV-26 test, while hyperactivity/impulsivity and oppositional defiance scores were at normal levels.

The Weiss Functional Impairment Rating Scales-Parent Report (WFIRS-P) is designed to assess the degree of impairment in the daily functioning of children and adolescents, encompassing areas such as family, school, life skills, self-management, social activities, and risky behaviors. The WFIRS-P assessment of the ADHD child revealed functional impairments across domains including family, school, life skills, and engagement in risky activities. Furthermore, the Child Behavior Check List (CBCL) categorizes behavioral problems into six primary behavioral symptom factors: social withdrawal, depression, sleep disturbances, somatic complaints, aggressive behavior, and destructive behavior. It aims to assess children's behavioral and emotional issues within the family, school, or community setting. The child's CBCL showed that she scored higher than normal in both social withdrawal and depression, implying that the child's performance in these specific domains tended to show symptoms of social withdrawal and depression more than most children of the same age. This may indicate that the child is having difficulty with social interactions, may feel isolated or unwilling to socialize with others, and may exhibit traits associated with depression in her emotional state, requiring close attention to the child's mental health.

The child's brain magnetic resonance imaging (MRI) at 9 months of age revealed delayed myelination maturation in comparison to peers. The most recent MRI of the brain revealed no anomalies. The child's blood routine, liver and kidney function, blood electrolytes, electrocardiogram, and electroencephalogram all show no abnormalities. Because the child had a history of recurrent cough, respiratory infections and wheezing, combined with allergic rhinitis, she needed to be alerted to the possibility of bronchial asthma. As a result, she underwent pulmonary ventilation testing that showed small airway airflow obstruction and impaired pulmonary ventilation.

## 4 Diagnostic inventory

The child was diagnosed with ADHD, cleft palate, VtSD, PFO, mild tricuspid regurgitation, delayed motor and language development, hearing loss, recurrent respiratory infections, asthma, rhinitis, enuresis, and dental caries based on her medical history, clinical symptoms, auxiliary examinations, and scale assessment results.

## 5 Patient's progress report

Given the clear diagnosis of ADHD in the child, she was treated with methylphenidate, starting at a dose of 18 mg/day, which was gradually increased to 36 mg/day based on her attentional performance, while also undergoing physical and language rehabilitation training. Additionally, the pediatrician advised the parents to engage the child in reading and retelling stories at home daily. The parents were fully involved in the treatment process. During the first year of treatment, the parents reported a positive impact, and the child's teacher noted an improvement in her attention span and the speed of completing homework. However, in the second year of treatment, both parents and the teacher reported that the child had difficulty concentrating again and exhibited learning difficulties, prompting an increase in the methylphenidate dose to 45 mg/day. This led to difficulties in falling asleep at night and poor sleep quality, so the dose was reduced back to 36 mg/day (Table 1). After two years of regular treatment, the child still met the diagnostic criteria for ADHD. Therefore, this suggests that methylphenidate was not effective in treating the child, and given her comorbid conditions, the possibility of inherited metabolic diseases should be considered. To further investigate the etiology and guide treatment, after obtaining informed consent from the parents, we conducted whole exome sequencing.

**Table 1:** Changes in methylphenidate dosage and attention deficit scores over time.

Times	Dosage (mg/day)	SNAP-IV-26 (attention deficit scores)
May 2022 to December 2022	18	17
January 2023 to February 2024	36	18
March 2024 to April 2024	45	21
May 2024 to August 2024	36	untested

(SNAP-IV-26) Swanson, Nolan, and Pelham, Version IV-26 items.

## 6 Results

WES was performed to further elucidate the causes of multiple diseases in our patient. A heterozygous frameshift mutation in the *MEIS2* gene (NM_170674.5: exon9: c.934_937del, p. Leu312Argfs*11) was identified through further genetic testing (Table 2). The *MEIS2* gene is a *de novo* mutation, which means it is not present in the genetic sequence of the parents but emerges as a new mutation in the child. This variant is the only *de novo* protein coding variant found in this case. It has been confirmed by Sanger sequencing. This variant has been reported in the study by Verheije et al. (26) and has (27) been recorded in the ClinVar database. Furthermore, according to ACMG criteria (28), the c.934_937del variant in this patient is classified as pathogenic.

**Table 2:** Mutation site details.

Gene name	Location	Gene mutation information	Mutation type	Variant classification (ACMG)	Disease	Zygosity type	Variant origin
*MEIS2*	Chr15:36950364_36950367	NM_170674.5: exon9: c.934_937del, p. Leu312Argfs*11	Frameshift mutation	Pathogenic	*MEIS2* syndrome	Heterozygote	*de novo*

## 7 Discussion

We report a case of *de novo* heterozygous frameshift mutation in the *MEIS2* gene that resulted in developmental delays and a phenotypic overlap of cardiac, craniofacial, and other abnormalities. The clinical presentation of this case included cleft palate, VtSD and PFO, ADHD, delayed motor and language development, mild facial dysmorphic features, learning difficulties, hearing loss, recurrent respiratory infections, asthma, rhinitis, enuresis, and dental caries. The clinical presentation of this patient shared some common clinical features with other previously described patients (21, 22, 26, 29–34), but also included some less common characteristics. Table 3 summarized the clinical phenotypes and genetic findings of this case and previously reported cases with *MEIS2* gene variants.

**Table 3:** The clinical phenotypes and genetic findings of this case and previously reported cases with *MEIS2* gene variants.

Publication	Case number	Sex	Age (yrs)	*MEIS2* variant	Cleft palate	Heart defects	Intellectual disability/delayed development	Dysmorphic features
Louw et al. (29)	1	F	5	c.998_1000del	CP	AtSD type II, VtSD, CA, LVOTO	Moderate ID	Bitemporal narrowing, arched and laterally extended eyebrows, mild upslanting palpebral fissures, deep-set eyes, a tented upper lip, thin upper vermilion, full lower vermilion, broad first ray of hands and feet, a gap between the first and second toes, and syndactyly of toe II–III
				p.Arg333del			Severely delayed gross motor, verbal development	
Fujita et al. (30)	1	F	2	c.611C > G	CP	AtSD, VtSD	Severe ID	Large forehead, mild trigonocephaly, sparse eyebrow, deeply set eyes, large and low-set ears, full cheeks and thin upper lip vermilion
				p.Ser204*			delayed motor development, speech delay	
Srivastava et al. (32)	1	F	NR	c.955A > G	BU	AtSD, VtSD	ID hypotonia	Minor dysmorphisms
				p.Arg319Gly				
Douglas et al. (31)	1	M	0.75	c.905C > T	BU	VtSD	ID	Micrognathia, full cheeks
				p.Pro302Leu			Developmental delay, fine and gross motor, speech	
	2	M	5	c.992G > A	CP, BU	VtSD	Severe ID	Broad face, full cheeks, plagiocephaly, deeply set eyes, eyelid ptosis, narrow nose with pointed tip, small nasal alae, short philtrum, eversion of lower lip, protruding ears with simple helix on right
				p.Arg331Lys			Global developmental delay, gross motor, speech and language delay	
	3	F	5	c.1004 T > C	CP	No	ID	NR
				p.Val335Ala			Global developmental delays, fine and gross motor, speech	
	4	F	12	c.965A > T	CP	No	Severe ID	Full lips, downturned corners of mouth, simple structure of ears, mild facial asymmetry
				p.Gln322Leu			Developmental delay, no verbal speech	
Verheije et al. (26)	1	M	5	c.978G > A	CP	VtSD	Mild ID	Mild eversion of the lower eyelids, fine arched eyebrows, and a small chin, prominent metopic suture
				p.Trp326*			Psychomotor development delay, fine and gross motor, speech	
	2	M	10	c.639 + 1G > A	CP	No	Mild/Moderate ID	Ptosis of the left eye, sagging of the lower eyelids, and square-shaped ear helices
				Splice variant			Developmental delay moderately delayed speech	
	3	M	4	c.640-2A > G	SMCP	No	Moderate ID	Epicanthic folds, hypoplastic alae nasi, prominent ears, and a frontal cow lick
				Splice variant			Developmental delay, motor and speech delay	
	4	F	20	c.829C > T	CP	Mitral valve insufficiency	Mild ID	Broad forehead with bitemporal narrowing
				p.Gln277*			Motor, cognitive and speech development delay	
	5	M	13	c.868dupA	BU	No	Mild ID	Prominent forehead, epicanthic folds, hypertelorism, long eyelashes with distichiasis, bulbous beaked nose with short alae nasi, thin upper lip, retrognathia, short neck, hypoplastic right nipple, and fifth finger clinodactyly, atypical medial eyebrow flare, micrognathia mild posterior rotation of ears
				p.Ile290Asnfs*40			Walk independently at 18 months old, speak at two years of age, speak sentences consisting of 2–3 words at three and a half years	
	6	F	5	c.978-2A > G	CP	No	Mild ID	Fine arched eyebrows and hypoplastic alae nasi
				Splice variant			Mild developmental delay, speech delay	
	7	F	18	c.383delA	NR	TOF Ebstein anomaly	No ID	No
				p.Lys128Serfs*19			Mild psychomotor developmental delay	
	8	F	11	c.934_937del	SMCP	AtSD type II, VtSD Ebstein anomaly	Mild ID	High and broad forehead, fine arched eyebrows, hypoplastic alae nasi, short philtrum, and low-set dysplastic ears with a right-sided ear pit, bilaterally overriding toes
				p.Leu312Argfs*11			Motor and speech development delay	
	9	M	1	c.998G > A	No	AtSD, LPVS, VtSD	Profound ID	High and small forehead with a high frontal hairline, frontal bossing and temporal narrowing, short palpebral fissures, hypertelorism, a depressed nasal bridge with anteverted nares, full cheeks, a smallmouth, short neck and limbs in comparison with the trunk
				p.Arg333Lys			Profound developmental delay	
Giliberti et al. (22)	1	M	8	c.520C > T	HP	AtSD, VtSD	ID	Large forehead, low frontal hairline, thick hair, thin and laterally extended eyebrows, large nasal tip with anteverted and hypoplastic nostrils, M-shaped upper lip, high palate, tapering fingers, sandal gap bilaterally
				p.Arg174*			Psychomotor and speech delay	
Santoro et al. (33)	1	M	15	c.27_28del	CP	No	Moderate-Severe ID	Brachycephaly, broad and sloping forehead, full eyes with upslanting palpebral fissures, thin upper vermilion, eyebrows widening medially and gradually thinning laterally, bulbous nasal tip and thin philtrum, upturned mouth corners, wide and lowly inserted columella, M-shaped/tented upper lip, and full lower vermilion, retrognathia, large and low-set ears, broad thumbs and great toes, scoliosis with sloping left shoulder, pectus excavatum, camptodactyly of toe II, and syndactyly of toes II–III
				p.His10Leufs*84			delayed psychomotor development	
Gangfuß et al. (21)	1	M	10	c.998G > A	CP	AtSD, PDA	ID	Thin and arched eyebrows, thin lip vermillion and prominent nasal tip with short alae nasi, and large, protruding, ears with enlarged fossa triangularis and hypoplastic antihelix
				p.Arg333Lys			Motor and speech development severely delayed	
Barili et al^.^ (34)	1	M	10	c.998G > A	CP	AtSD, TOF	Moderate-Severe ID	Finely arched eyebrows,broad forehead, moderately shortened philtrum and tented upper lip
				p.Arg333Lys			delayed psychomotor development absent speech at 5 years of age	
Current case	1	M	10	c.934_937del	CP	VtSD, PFO	No ID	Broad forehead, elongated and arched eyebrows, tent-shaped upper lip
				p.Leu312Argfs*11			Developmental delay, fine and gross motor, speech	

Publication	Case Number	Behavioral problems	Other features
Louw et al. (29)	1	ASD	Congenital lobar emphysema, severe feeding problems (gastro-esophageal reflux, oral aversion, aerophagia, and achalasia)
Fujita et al. (30)	1	No	Serious feeding difficulty with severe gastro-esophageal reflux, severe hypermetropia, severe constipation
Srivastava et al. (32)	1	ASD	Stereotyped hand movements and impaired sleep pattern, seizures, bruxism when awake, intense eye communication
Douglas et al. (31)	1	No	Delayed myelination, Bilateral conductive hearing loss, short stature, failure to thrive, poor weight gain, mild hypotonia, sacral dimple, dermatitis
	2	Autism	Staring spells, dysphagia, drooling, chewing difficulties, low facial muscle tone, choking, vomiting, epigastric hernia, hypotonia, broad thorax with widely spaced, inverted nipples, elongated tailbone, genu valgum, toe-walking, wide based gait, with poor coordination, scoliosis, based gait, with poor coordination, scoliosis, long, tapered fingers, deep set nails, puffy hands, planovalgus, sacral dimple, dermatitis, mild eczema
	3	ASD, short attention span, stereotypic behavior	Prominence of CSF spaces, hypotonia, hypertonia and spasticity in legs leading to abnormal gait, hyperextensible joints
	4	Autism, self-injurious and repetitive behaviors (clapping, hand flapping, chest thumping)	Febrile, grand mal, drop seizures, staring spells, hearing improved with myringotomies, chronic constipation, facial muscle hypotonia
Verheije et al. (26)	1	No	No
	2	ASD (temper tantrums, aggressive behavior, and short attention span)	Gastro-esophageal reflux, conductive hearing loss, sleep apnea, mild asymmetry of the lateral ventricles
	3	ASD	Poor suck, hypermetropia, strabismus, cryptorchidism, and difficulties with co-ordination
	4	No	Limited movement with intermittent hyperextension of the trunk, mild myopia with adequate vision, intermittent hearing impairment, minimal billowing and insufficiency of the mitral valve
	5	No	Cryptorchidism, retractile right testicle, phimosis/meatal stenosis rep, precocious adrenarche and evaluated, borderline/low testosterone levels after stimulation, recurrent ear infections and possible hearing loss, ocular melanocytosis and iris nevus, pre-glaucoma
	6	No	Learning difficulties
	7	No	Feeding difficulties
	8	No	Severe feeding problems
	9	Restless behavior	Duodenal stenosis, bilateral inguinal hernia, permanent respiratory insufficiency, hypothyroidism, transitory pancreatitis, nephrocalcinosis, feeding difficulties, recurrent vomiting, failure to thrive, high temperatures without infections, bilateral ventriculomegaly and brain atrophy, severe muscular hypotonia of the trunk and hypertonia of the limbs, poor head control, cannot roll over, cannot interact and grasp, strained breathing
Giliberti et al. (22)	1	ASD	Undescended right testicle, stereotypic hand and trunk movements, ectasia and gliosis of Virchow-Robin areas
Santoro et al. (33)	1	No	Minimal mitral regurgitation, brain MRI revealed the presence of UBOs and hypoplasia of the corpus callosum, cafe-au-lait spots, inguinal freckling and cutaneous neurofibromas, severe constipation, CT scan highlighted the early partial closure of both coronal cranial sutures
Gangfuß et al. (21)	1	ASD, (auto-)aggression, lack of distance, aggressive behavior	Feeding difficulties, tracheomalacia (including stenosis of left main bronchus), recurrent pulmonary infections, dystrophy, muscular hypotonia, hypermobile joints, and a mild right convex thoracic scoliosis
Barili et al. (34)	1	NR	Brain anomalies
Current case	1	ADHD	Poor coordination, mild tricuspid regurgitation, recurrent respiratory infections, asthma, rhinitis, enuresis, dental caries, hearing loss due to otitis media, learning difficulties, brain MRI showed a delayed level of mature myelination, moderately impaired lung function

ASD, Autism spectrum disorder; F, Female; M, Male; yrs, Years; NR, No report; BU, Bifid uvula; SMCP, Submucous cleft palate; AtSD, Atrial septal defect; VtSD, Ventricular septal defect; CA, Coarctation of the aorta; LVOTO, Left ventricular outflow tract obstruction; CSF, Cerebrospinal fluid; LPVS, Left pulmonary vein stenosis; TOF, Tetralogy of Fallot; PFO, Patent foramen ovale; PDA, Patent ductus arteriosus; MRI, Magnetic resonance imaging; UBOs, Unidentified bright objects; CT, Computerized tomography.

The diagnosis and treatment of ADHD are further complicated by the fact that ADHD often co-exists with other disorders such as learning difficulties, anxiety disorders, and oppositional defiant disorder. Currently, the exact etiology of ADHD remains undetermined, but researchers generally agree that ADHD is the result of a combination of genetic and environmental factors (35, 36). With the advent of innovative genomics technologies, genetic evaluation of patients with complex clinical presentations has become possible. We report the patient diagnosed with ADHD, who also has a variety of other diseases, presenting with complex clinical manifestations. In addition, her treatment with methylphenidate showed no significant improvement in attention deficit. In search of the etiology, she underwent WES, which revealed a mutation in the *MEIS2* gene. This further illustrates that genetic testing is an important clinical tool for identifying the causes in ADHD patients with multiple comorbidities and poor response to methylphenidate treatment.

To date, a total of 21 patients carrying *MEIS2* gene mutations have been reported, with 20 patients detected through whole-exome sequencing, and only one patient in the study by Verheije et al. (26) identified using targeted sequencing. This suggests that the phenotype associated with *MEIS2* gene mutations is not easily recognizable. Among the reported patients, most of them had intellectual disability, oral-facial clefts (18/21), similar facial malformation characteristics (19/21), and developmental delay (20/21), suggesting that intellectual disability, oral-facial clefts, facial malformations, and developmental delay are common characteristics. However, the patient we reported did not have an intellectual disability. Approximately half of the patients have cardiac defects (14/21), with ventricular septal defect being the most common feature. However, in three cases, more severe cardiac defects were reported, including CA, TOF, and Ebstein's anomaly. This is because these three severe cases carried a frameshift mutation or an intronic deletion of a single highly conserved amino acid (Arg333) (Patient by Louw et al. and Patients 7 and 8 by Verheije et al.) (26, 29). Our patient shared the same *MEIS2* gene frameshift mutation with the patient 8 reported by Verheije et al. (26), yet the cardiac defects manifested only as a mild VtSD and PFO, with the ventricular septal defect resolving spontaneously over time.

ASD has been identified in some of the patients with *MEIS2* gene mutations that have been documented thus far (9/21). But attention deficits are uncommon in patients with the *MEIS2* gene mutation. The patient in this case report had attention deficits that met the diagnostic criteria for ADHD, a diagnosis that had never before been made in a case with a mutation in the *MEIS2* gene. However, two additional patients with *MEIS2* gene mutations have shown comparable results. Douglas et al. (31) and Verheije et al. (26) reported a 5-year-old girl and a 10-year-old boy, respectively, both diagnosed with ASD and showing short attention spans. Overall, more patients carrying *MEIS2* gene mutations are needed to better define the genotype-phenotype correlations.

Recently, Verheije et al. (26) described a patient with the same frameshift mutation (c.934_937del, p. Leu312Argfs*11). This patient had right ventricular hypoplasia, VtSD, PFO, and tricuspid valve abnormalities, which resulted in severe cyanosis at birth. She also had severe eating issues, delayed motor and language development, a moderate intellectual handicap, a cleft soft palate, and a slight indentation of the hard palate, as well as facial malformations. This patient and the case we reported had comparable clinical symptoms, including delayed motor and verbal development, cleft palate, VtSD, PFO, and facial malformations. In addition, our patient had ADHD, recurrent respiratory infections, learning difficulties, hearing loss, asthma, rhinitis, enuresis, and dental caries. However, she did not exhibit intellectual disability, which further expands the spectrum of clinical phenotypes caused by the same *MEIS2* gene mutation.

Research has demonstrated that the human, mouse, and chicken *MEIS2* gene regulates specific areas of the developing brain, indicating its significance in neurocognitive development (27, 37, 38). In chicken and mouse heart tissue, DeLaughter et al. (39) showed how the *MEIS2* gene contributes to the conversion of endothelial cells to endocardial mesenchyme. While research indicates that *MEIS2* gene knockout mice are embryonically deadly, conditional knockout mice display aberrant development of the heart, cranial nerves, and craniofacial bones (7), providing evidence to further support that a phenotype of general developmental abnormalities was caused by a disruption of normal human *MEIS2* gene function. Furthermore, research has shown a connection between the *MEIS2* gene and neurodegenerative illnesses and intellectual disability (40, 41).

Additionally, research has demonstrated that the *MEIS2* gene has a role in the etiology of human malignancies (42, 43). Abnormal expression of *MEIS2* significantly impacts neuroblastoma cell proliferation and tumorigenicity (44). Wan et al. (45) first demonstrated that *MEIS2* acts as a metastasis promoter in colorectal cancer. Xiao et al. (46) found that *MEIS2* functions as a tumor suppressor in breast cancer development. A recent study revealed that targeting *MEIS2* expression inhibits proliferation and invasion of prostate cancer cells (47). High levels of *MEIS2* are correlated with poor survival rates in patients with liver cancer (48). *MEIS2* expression is highly downregulated in thyroid cancer patients, as demonstrated by Wen et al. (49) suggesting that *MEIS2* may be a target for early diagnosis and targeted therapy in these individuals. *MEIS2* inhibits the expression of genes specific to the ciliary marginal zone and optic disc and increases the expression of genes specific to retinal progenitor cells (16). *MEIS2* plays a role in acute myeloid leukemia (AML)-ETO-positive leukemia (50). Conversely, high expression of *MEIS2* is associated with improved prognosis in ovarian cancer patients (51). Therefore, *MEIS2* can be considered as one of the genes involved in neurodevelopmental disorders and cancers, such as those related to the RAS pathway genes or BAF complex genes (37, 52).

Our report demonstrates the importance of genetic testing to find the etiology of ADHD in patients with multiple comorbidities who are poorly treated with methylphenidate. In addition, this case further extends the clinical phenotype of mutations in the *MEIS* gene.

## Data Availability

The original contributions presented in this study are included in this article/Supplementary material, further inquiries can be directed to the corresponding author.
